# Tensor methods for parameter estimation and bifurcation analysis of stochastic reaction networks

**DOI:** 10.1098/rsif.2015.0233

**Published:** 2015-07-06

**Authors:** Shuohao Liao, Tomáš Vejchodský, Radek Erban

**Affiliations:** 1 Mathematical Institute, University of Oxford, Radcliffe Observatory Quarter, Woodstock Road, Oxford OX2 6GG, UK; 2 Institute of Mathematics, Czech Academy of Sciences, Zitna 25, 115 67 Praha 1, Czech Republic

**Keywords:** gene regulatory networks, stochastic modelling, parametric analysis, high-dimensional computation

## Abstract

Stochastic modelling of gene regulatory networks provides an indispensable tool for understanding how random events at the molecular level influence cellular functions. A common challenge of stochastic models is to calibrate a large number of model parameters against the experimental data. Another difficulty is to study how the behaviour of a stochastic model depends on its parameters, i.e. whether a change in model parameters can lead to a significant qualitative change in model behaviour (bifurcation). In this paper, tensor-structured parametric analysis (TPA) is developed to address these computational challenges. It is based on recently proposed low-parametric tensor-structured representations of classical matrices and vectors. This approach enables simultaneous computation of the model properties for all parameter values within a parameter space. The TPA is illustrated by studying the parameter estimation, robustness, sensitivity and bifurcation structure in stochastic models of biochemical networks. A Matlab implementation of the TPA is available at http://www.stobifan.org.

## 1. Introduction

Many cellular processes are influenced by stochastic fluctuations at the molecular level, which are often modelled using stochastic simulation algorithms (SSAs) for chemical reaction networks [1,2]. For example, cell metabolism, signal transduction and cell cycle can be described by network structures of functionally separated modules of gene expression [3], the so-called gene regulatory networks (GRNs).

Typical GRN models can have tens of variables and parameters. Traditionally, GRNs have been described using continuous deterministic models written as systems of ordinary differential equations (ODEs). Several methodologies for studying parametric properties of ODE systems, such as identifiability and bifurcation, have been developed in the literature [4–8]. Recently, experimental evidence has highlighted the significance of intrinsic randomness in GRNs, and stochastic models have been increasingly used [1,9]. They are usually simulated using the Gillespie SSA [10], or its equivalent formulations [11,12]. However, methods for parametric analysis of ODEs cannot be directly applied to stochastic models. In this paper, we present a *tensor-structured parametric analysis* (TPA) which can be used to understand how molecular-level fluctuations influence the system-level behaviour of GRNs and its dependence on model parameters. We illustrate major application areas of the TPA by studying several biological models with increasing level of complexity.

The parametric analysis of GRN models is computationally intensive because both state space and parameter space are high-dimensional. The dimension of the state space, *Ω*_**x**_, is equal to the number of reacting molecular species, denoted by *N*. When an algorithm, previously working with deterministic steady states, is extended to stochastic setting, its computational complexity is typically taken to the power *N*. Moreover, the exploration of the parameter space, *Ω***_k_**, introduces another multiplicative exponential complexity. Given a system that involves *K* parameters, the ‘amount’ of parameter combinations to be characterized scales equally with the volume of *Ω***_k_**, i.e. it is taken to the power *K* [13].

The TPA framework avoids the high computational cost of working in high-dimensional *Ω*_**x**_ and *Ω***_k_**. The central idea is based on generalizing the concept of separation of variables to parametric probability distributions [14]. The TPA framework can be divided into two main steps: a tensor-structured computation and a tensor-based analysis. First, the steady-state distributions of stochastic models are simultaneously computed for all possible parameter combinations within a parameter space and stored in a tensor format, with smaller computational and memory requirements than in traditional approaches. The resulting tensor data are then analysed using algebraic operations with computational complexity which scales linearly with dimension (i.e. linearly with *N* and *K*).

The rest of this paper is organized as follows. In §2, we discuss how the parametric steady-state probability distribution can be presented and computed in tensor formats. We illustrate the data storage savings using tensor-structured simulations of four biological systems. The stored tensor data are then used as the input for the tensor-based analysis presented in the subsequent sections. In §3, we show that the existing procedures for parameter inference for deterministic models can be directly extended to the stochastic models using the computed tensor data. In §4, a direct visualization of stochastic bifurcations in a high-dimensional state space is presented. The TPA of the robustness of the network to extrinsic noise is illustrated in §5. We conclude with a brief discussion in §6.

## 2. Tensor-structured computations

Considering a well-mixed chemically reacting system of *N* distinct molecular species *X_i_*, *i* = 1, 2, … , *N*, inside a reactor (e.g. cell) of volume *V*, we denote its state vector by 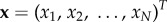, where *x_i_* is the number of molecules of the *i*th chemical species *X_i_*. In general, the volume *V* can be time dependent (for example, in cell cycle models which explicitly take into account cell growth), but we will focus in this paper on models with constant values of *V*. We assume that molecules interact through *M* reaction channels2.1
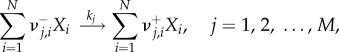
where 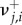 and 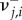 are the stoichiometric coefficients. The kinetic rate parameters, 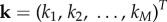, characterize the rate of the corresponding chemical reactions. We will treat **k** as auxiliary variables, and, in other words, the parametric problem of (2.1) involves considering both 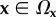 and 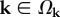. In this paper, we study problems where the dimension of the parameter space *K* is equal to *M*. We also consider cases where some rate constants are not varied in the parameter analysis, i.e. *K* < *M*. In this case, notation **k** will be used to denote *K*-dimensional vector of rate constants, 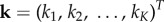, which are considered during the TPA. The values of the remaining (*M* − *K*) rate constants are fixed. In principle, the TPA could also be used to study models where *K* > *M*, i.e. when we consider additional parameters (e.g. system volume *V*).

Let 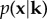 be the steady-state probability distribution that the state vector is **x** (if the system is observed for sufficiently long time) given the parameter values **k**. The main idea of the TPA is to split 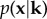 in terms of coordinates as2.2
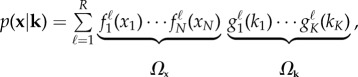
where 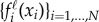 and 
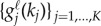
 are univariate functions that vary solely with a single state variable and parameter, respectively. The number of summands *R*, the so-called *separation rank*, controls the accuracy of the decomposition (2.2). By increasing *R*, the separated expansion could theoretically achieve arbitrary accuracy.

The value of the separation rank *R* can be analytically computed for simple systems. For example, there are analytical formulae for the stationary distributions of first-order stochastic reaction networks [15]. They are given in the form (2.2) with *R* = 1. Considering second-order stochastic reaction networks, there are no general analytical formulae for steady-state distributions. They have to be approximated using computational methods. The main assumption of the TPA approach is that the parametric steady-state distribution has a sufficiently accurate low-rank representation (2.2). In this paper, we show that this assumption is satisfied for realistic biological systems by applying the TPA to them and presenting computed (converged) results. The main consequence of low-rank representation (2.2) is that mathematical operations on the probability distribution 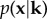 in *N* + *K* dimensions can be performed using combinations of one-dimensional operations, and the storage cost is bounded by (*N* + *K*)*R*. The rank *R* may also depend on *N* + *K* and the size of the univariate functions in (2.2). Numerical experiments have shown a linear growth of *R* with respect to *N* + *K* and a logarithmic growth with respect to the size of the univariate functions in the representation (2.2) [16,17]. To find the representation (2.2), we solve the chemical Fokker–Planck equation (CFPE), as a (fully) continuous approximation to a (continuous time) discrete space Markov chain described by the corresponding chemical master equation (CME) [18,19]. Specifically, we keep all the objects in the separated form of (2.2) during the computations, such that exponential scaling in complexity does not apply during any step of the TPA.

We refer to the representation (2.2) as tensor-structured, because computations are performed on 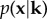 as multi-dimensional arrays of real numbers, which we call tensors [20]. The (canonical) tensor decomposition [21], as a discrete counterpart of (2.2), then allows a multi-dimensional array to be approximated as a sum of tensor products of one-dimensional vectors. Within such a format, we can define standard algebraic operations similar to standard matrix operations such that the resulting tensor calculus enables efficient computation. The tensor-structured parametric steady-state distribution (2.2) is approximated as the eigenfunction corresponding to the smallest eigenvalue of the parametric Fokker–Planck operator. The operator is constructed in a tensor separated representation as a sum of tensor products of one-dimensional operators. The eigenfunction is computed by the adaptive shifted inverse power method, using the minimum alternating energy method as the linear solver. We leave further discussion of technical computational details of the underlying methods to electronic supplementary material, appendix S1. The TPA has been implemented in Matlab and is part of the Stochastic Bifurcation Analyzer toolbox available at http://www.stobifan.org. The source code relies on the Tensor Train Toolbox [22].

### 2.1. Applications of the tensor-structured parametric analysis to biological systems

We demonstrate the capabilities of the TPA framework by investigating four examples of stochastic reaction networks: a bistable switch in the five-dimensional Schlögl model [23], oscillations in the seven-dimensional cell cycle model [24], neurons excitability in the six-dimensional FitzHugh–Nagumo system [4] and a 20-dimensional reaction chain [25] (see electronic supplementary material, appendix S2, for more details of these models). Table 1 compares computational performance of the TPA with the traditional matrix-based methods for the computation of the parametric steady-state distribution 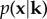. The minimum memory requirements of solving the CME and the CFPE using matrix-based methods, Mem_CME_ and Mem_CFPE_, are estimated as products of numbers of discrete states times the total number of parameter combinations. They vary in ranges 10^13^–10^44^ and 10^11^–10^54^, respectively, which are beyond the limits of the available hardware. In contrast, the TPA maintains affordable computational and memory requirements for all four problems considered, as we show in table 1. The major memory requirements of the TPA are Mem_A_ and Mem*_p_* to store the discretized Fokker–Planck operator and the steady-state distribution *p*(**x**|**k**), respectively (see electronic supplementary material, appendix S1, for detailed definitions). Similarly, *T***_A_** is the computational time to assemble the operator and *T*_tot_ is the total computational time.

**Table 1.** Comparison of the matrix-based and tensor-structured methodologies.

biochemical system	dimensionality			matrix-based^a^		tensor-structured^b^			
	*N*	*K*	*N* + *K*	Mem_CME_	Mem_CFPE_	Mem_A_	Mem*_p_*	*T*_A_ (s)	*T*_tot_ (min)
Schlögl	1	4	5	2.68 × 10^13^	2.74 × 10^11^	4.01 × 10^3^	2.07 × 10^5^	1.2	30
cell cycle	6	1	7	6.68 × 10^17^	7.04 × 10^13^	2.96 × 10^4^	1.00 × 10^7^	1.1	6433
FitzHugh–Nagumo	2	4	6	6.38 × 10^14^	1.75 × 10^13^	7.65 × 10^4^	4.02 × 10^5^	0.7	37
reaction chain	20	0	20	1.20 × 10^44^	1.53 × 10^54^	9.26 × 10^4^	7.28 × 10^5^	15.6	283

^a^Estimated as the product of the number of discrete states and the number of parameter values.

^b^Mem**_A_** is the storage requirement for the discrete Fokker–Planck operator in tensor structure. It can be avoided by computing all matrix–vector products on the fly.

Table 1 shows that the TPA can outperform standard matrix-based methods. It can also be less computationally intensive than stochastic simulations in some cases. For example, the total computational time is around 30 min for the TPA to simulate 64^4^ different parameter combinations within the four-dimensional parameter space of the Schlögl chemical system (table 1). If we wanted to compute the same result using the Gillespie SSA, we would have to run 64^4^ different stochastic simulations. If they had to be all performed on one processor in 30 min, then we would only have 1.07 × 10^−4^ s per one stochastic simulation and it would not be possible to estimate the results with the same level of accuracy. In addition, the TPA directly provides the steady-state distribution *p*(**x**|**k**), which would be computationally intensive to obtain by stochastic simulations (with the same level of accuracy) for larger values of *N* + *K*.

## 3. Parameter estimation

Small uncertainties in the reaction rate values of stochastic reaction networks (2.1) are common in applications. Some model parameters are difficult to measure directly, and instead are estimated by fitting to time-course data. If GRNs are modelled using deterministic ODEs, there is a wide variety of tools available for parameter estimation. Many simple approaches are non-statistical [26], and the procedure usually, although not necessarily [27], follows the algorithm presented in table 2. This approach seeks the set of those parameters that minimize the distance measure 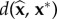, while the rules to generate candidate parameters k* in step (a1) and the definition of distance function along with stopping criteria in step (a3) may vary in different methods. In optimization-based methods, k* may follow the gradient on the surface of the distance function [26]. In statistical methods, such distance measure is provided in the concept of likelihood, 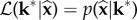 [28]. In Bayesian methods, the candidate parameters k* are generated from some prior information regarding uncertain parameters, 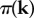, and form a posterior distribution rather than a single point estimate [29].

**Table 2.** Parameter estimation for ODEs.

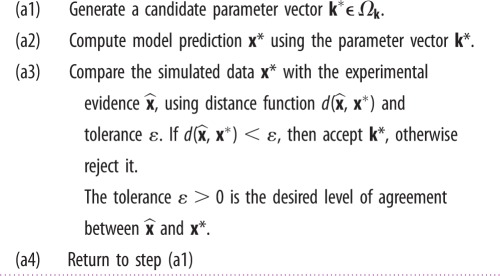

Extending the algorithm in table 2 from deterministic ODEs to stochastic models requires substantial modifications [29]. One main obstacle is the step (a2) which requires repeatedly generating the likelihood function 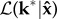, as the outcome of stochastic models. In this case, a modeller must either apply statistical analysis to approximate the likelihood [30], or use the Gillespie SSA to estimate it [31]. Consequently, the algorithms are computationally intensive and do not scale well to problems of realistic size and complexity. To avoid this problem, the TPA uses the tensor formalism to separate the simulation part from the parameter inference. The parameter estimation is performed on the tensor data obtained by methods described above (table 1). The algorithm used for the TPA parameter estimation is given in table 3. The distance function 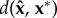 is replaced with a distance between summary statistics, 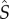 and S*, which describe the average behaviour and the characteristics of the system noise. The steps (b1), (b3) and (b4) are similar to steps (a1), (a3) and (a4) under the ODE settings, and a variety of existing methods can be extended directly to stochastic settings. The newly introduced step (b0) is executed only once during the parameter estimation. Steps (b1)–(b4) are then repeated until convergence. Step (b2) only requires manipulation of tensor data, of which the computational overhead is comparable to solving an ODE.

**Table 3.** An algorithm for the tensor-structured parameter estimation.

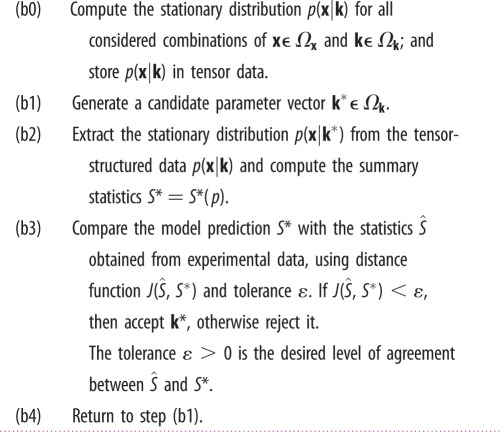

### 3.1. An example of parameter estimation

We consider that the distance measure 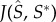 in table 3 is defined using a moment-matching procedure [32,33]:3.1
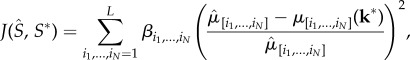
where 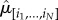 is the (*i*_1_,…,*i*_*N*_)th order empirical raw moment, 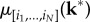 is the corresponding moment derived from 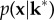 and *L* denotes the upper bound for the moment order. The weights, 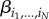, can be chosen by modellers to attribute different relative importances to moments. Empirical moments are estimated from samples 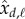, *d* = 1,2,…,*N*, 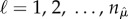, by3.2
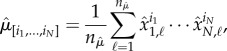
where 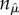 is the number of samples. Moments of the model output are computed as3.3
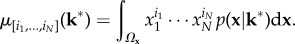
We show, in electronic supplementary material, appendix S1.4, that it is possible to directly compute different orders of moments, 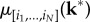, using the representation (2.2) with *O*(*N*) complexity.

**Figure 1.**
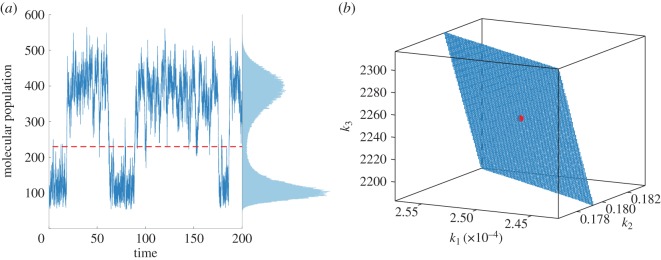
(*a*) A short segment of the time-series data and the histogram for the Schlögl reaction system generated by a long-time stochastic simulation. The dashed line corresponds to the threshold 230 which is used to separate the two macroscopic states of this bistable system. (*b*) The triples of parameters [*k*_1_, *k*_2_, *k*_3_] for which the splitting probability (3.4) is equal to 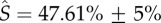 form a plane with a very thin thickness (in blue) within the three-dimensional parameter space. The value of *k*_4_ is fixed at its true value, and the true value of [*k*_1_, *k*_2_, *k*_3_] is marked with the red dot. (Online version in colour.)

**Figure 2.**
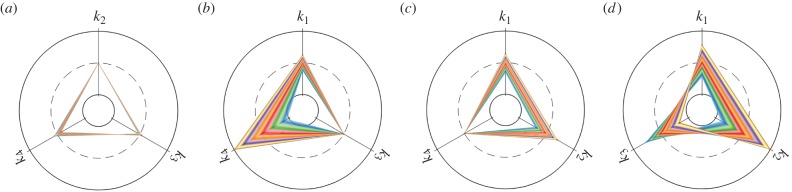
Circular representation [34] of estimated parameter combinations for the Schlögl model. Each spoke represents the corresponding parameter range listed in electronic supplementary material, table S3. The true parameter values are specified by the intersection points between the spokes and the dashed circle. Each triangle (or polygon in general) of a fixed colour corresponds to one admissible parameter set with *ɛ* = 0.25%. Each panel (*a*–*d*) shows the situation with one parameter fixed at its true value, namely (*a*) *k*_1_ is fixed; (*b*) *k*_2_ is fixed; (*c*) *k*_3_ is fixed; and (*d*) *k*_4_ is fixed.

We illustrate the tensor-structured parameter estimation using the Schlögl chemical system [23], which is written for *N* = 1 molecular species and has *M* = 4 reaction rate constants *k_i_*, *i* = 1, 2, 3, 4. A detailed description of this system is provided in electronic supplementary material, appendix S2.1. We prescribe true parameter values as *k*_1_ = 2.5 × 10^−4^, *k*_2_ = 0.18, *k*_3_ = 2250 and *k*_4_ = 37.5, and use a long-time stochastic simulation to generate a time series as pseudo-experimental data (for a short segment, see figure 1*a*). These pseudo-experimental data are then used for estimating the first three empirical moments 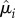, *i* = 1, 2, 3, using (3.2). While the moments of the model output, 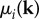, *i* = 1, 2, 3, are derived from the tensor-structured data *p*(**x**|**k**), computed using (2.2). Moment matching is sensitive to the choice of weights [33]. However, for the sake of simplicity, we choose the weights *β*_*i*_, *i* = 1, 2, 3, in a way that the contributions of the different orders of moments are of similar magnitude within the parameter space. Having the stationary distribution stored in the tensor format (2.2), we can then efficiently iterate steps (b1)–(b4) in table 3 to search for parameter values that produce adequate fit to the samples using the measure given in equation (3.1). We consider *ɛ* = 0.25% and visualize in figure 2 the admissible parameter values satisfying 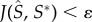.

The summary statistics 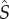 are not restricted to lower order moments. The TPA can efficiently evaluate different choices of the summary statistics, because of the simplicity and generality of separable representation (2.2). For example, if one can experimentally measure the probability that the system stays in each of the two states of the bistable system, then distance measure 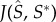 can be based on the probability of finding the system within a particular part of the state space *Ω*_**x**_. We show, in electronic supplementary material, appendix S1.4, that such quantity can also be estimated in the tensor format efficiently with *O*(*N*) complexity. Considering the Schlögl model, we estimate the probability that the system stays in the state with less molecules by3.4
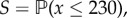
where ℙ denotes the probability and the threshold 230 separates the two macroscopic states of the Schlögl system, see the dashed line in figure 1*a*. The splitting probability (3.4) can be estimated using long-time simulation of the Schlögl system as the fraction of states which are less or equal than 230 and is equal to 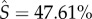 for our true parameter values. Figure 1*b* shows the set of admissible parameters within the parameter space *Ω*_**k**_ whose values provide desired agreement on the splitting probability (3.4) with tolerance *ɛ* = 5%, i.e. we use
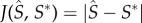
in the algorithm given in table 3, where S* is computed using (2.2) and (3.4).

### 3.2. Identifiability

**Figure 3.**
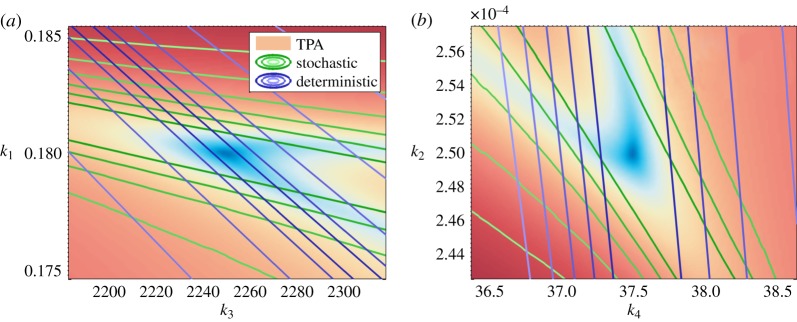
Parameter identifiability analysis of the Schlögl reaction system. (*a*) Estimation and identifiability of parameters *k*_1_ and *k*_3_ with *k*_2_ and *k*_4_ fixed at their true values. The colour scale corresponds to values of the distance function (3.1) with *L* = 3, i.e. the first three moments are compared. The green and blue contour lines indicate the distance functional (3.1) with the first moment (mean value) only. Green corresponds to the stochastic model and blue to the deterministic model. (*b*) Estimation and identifiability of parameters *k*_2_ and *k*_4_ with *k*_1_ and *k*_3_ fixed at their true values. The same quantities as in panel (*a*) are plotted.

One challenge of mathematical modelling of GRNs is whether unique parameter values can be determined from available data. This is known as the problem of identifiability. Inappropriate choice of the distance measure may yield ranges of parameter values with equally good fit, i.e. the parameters being not identifiable [35]. Here, we illustrate the tensor-structured identifiability analysis of the deterministic and stochastic models of the Schlögl chemical system. We plot the distance function against two parameter pairs, rate constants *k*_1_–*k*_3_ and *k*_2_–*k*_4_, in figure 3. From the colour map, we see that the distance function (3.1) possesses a well distinguishable global minimum at the true values (*k*_1_ = 2.5 × 10^−4^, *k*_2_ = 0.18, *k*_3_ = 2250 and *k*_4_ = 37.5). This indicates that the stochastic model is identifiable in both cases. In the deterministic scenario, the Schlögl system loses its identifiability. When the distance function (3.1) only fits the mean concentration, the minimal values are attained on a curve in the two-dimensional parameter space (the distance function is indicated by blue contour lines in figure 3). Stochastic models are advantageous in model identifiability, because they can be parametrized using a wider class of statistical properties (typically, *K* quantities are needed to estimate *K* reaction rate constants for mass–action reaction systems). The TPA enables efficient and direct evaluation of 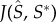 all over the parameter space in a single computation by using the representation (2.2).

Figure 3 also reveals the differences between the model responses to parameter perturbations. The green contour lines show the landscape of 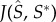 for the stochastic model using only the mean values, i.e. *L* = 1 in (3.1). The minimum is attained on a straight line, representing another non-identifiable situation. This line (green) has a different direction than the line obtained for the deterministic model (blue). In particular, this example illustrates that the parameter values estimated from deterministic models do not give good approximation of both average behaviour and the noise level when they are used in stochastic models [36].

## 4. Bifurcation analysis

**Figure 4.**
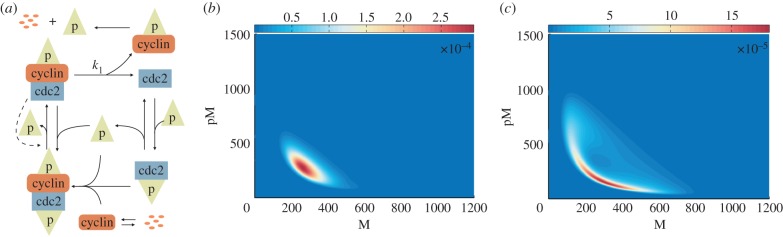
Bifurcation analysis of the stochastic cell cycle model. (*a*) Schematic description of the cyclin–cdc2 interactions. Free cyclin molecules combine rapidly with phosphorylated cdc2, to form the dimer MPF (cdc2–cyclin–p), which is immediately inactivated by phosphorylation process. The inactive MPF (p–cdc2–cyclin–p) can be converted to active MPF by autocatalytic dephosphorylation. The active MPF in excess breaks down into cdc2 molecules and phosphorylated cyclin, which is later subject to proteolysis. Finally, cdc2 is phosphorylated to repeat the cycle. (*b*) Joint stationary distribution of cdc2–cyclin–p (M) and p–cdc2–cyclin–p (pM) plotted at the deterministic bifurcation point (*k*_1_ = 0.2694). (*c*) Joint stationary distribution of cdc2–cyclin–p (M) and p–cdc2–cyclin–p (pM) plotted for *k*_1_ = 0.3032.

Bifurcation is defined as a qualitative transformation in the behaviour of the system as a result of the continuous change in model parameters. Bifurcation analysis of ODE systems has been used to understand the properties of deterministic models of biological systems, including models of cell cycle [37] and circadian rhythms [38]. Software packages, implementing numerical bifurcation methods for ODE systems, have also been presented in the literature [39,40], but computational methods for bifurcation analysis of corresponding stochastic models are still in development [19]. Here, we use the tensor-structured data 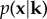 given by (2.2) for a model of fission yeast cell cycle control developed by Tyson [24], and perform the tensor-structured bifurcation analysis on the tensor data. The interaction of cyclin–cdc2 in the Tyson model is illustrated in figure 4*a*. Reactions and parameter values are given in electronic supplementary material, appendix S2.2.

The parameter *k*_1_, indicating the breakdown of the active M-phase-promoting factor (MPF), is chosen as the bifurcation parameter. The analysis of the corresponding ODE model reveals that the system displays a stable steady state when *k*_1_ is at its low values, which describes the metaphase arrest of unfertilized eggs [41]. On the other hand, the ODE model is driven into rapid cell cycling exhibiting oscillations when *k*_1_ increases [24]. The ODE cell cycle model has a bifurcation point at *k*_1_ = 0.2694, where a limit cycle appears [24]. In our TPA computations, we study the behaviour of the stochastic model for the values of *k*_1_ which are close to the deterministic bifurcation point. We observe that the steady-state distribution changes from a unimodal shape (figure 4*b*) to a probability distribution with a ‘doughnut-shaped’ region of high probability (figure 4*c*) at *k*_1_ = 0.3032. In particular, the stochastic bifurcation appears for higher values of *k*_1_ than the deterministic bifurcation.

**Figure 5.**
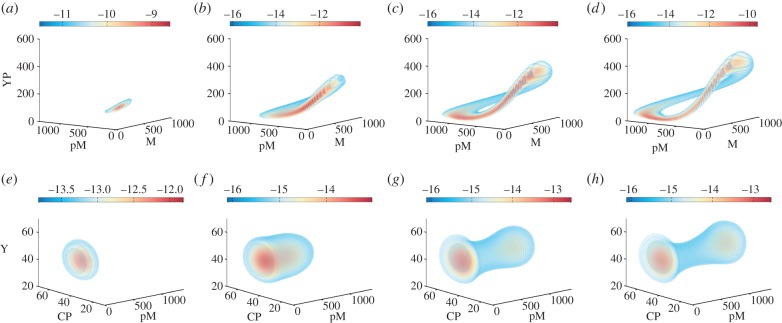
Visualization of the bifurcation structure of the stochastic cell cycle model. (*a*–*d*) Marginal steady-state distributions of the phosphorylated cyclin (YP), the inactive MPF (pM) and the active MPF (M). (*e*–*h*) Marginal stationary distributions of the cyclin (Y), the phosphrylated cdc2 (CP) and the inactive MPF (pM). Each column corresponds to the same value of the bifurcation parameter *k*_1_, which from the left to the right are 0.24, 0.3, 0.35 and 0.4, respectively. All figures show log_2_ of the marginal steady-state distribution for better visualization.

In figure 5, we use the computed tensor-structured parametric probability distribution to visualize the stochastic bifurcation structure of the cell cycle model. As the bifurcation parameter *k*_1_ increases, the expected oscillation tube is formed and amplified in the marginalized YP-pM-M state space (figure 5*a*–*d*). In figure 5*e*–*h*, the marginal distribution in the Y-CP-pM subspace is plotted. We see that it changes from a unimodal (figure 5*e*) to a bimodal distribution (figure 5*f*). Cell cycle models have been studied in the deterministic context either as oscillatory [24] or bistable [42,43] systems. In figure 5, we see that the presented stochastic cell cycle model can appear to have both oscillations and bimodality, when different subsets of observables are considered.

## 5. Robustness analysis

GRNs are subject to extrinsic noise which is manifested by fluctuations of parameter values [44]. This extrinsic noise originates from interactions of the modelled system with other stochastic processes in the cell or its surrounding environment. We can naturally include extrinsic fluctuations under the tensor-structured framework. For a GRN as in (2.1), we consider the copy numbers *X*_1_, *X*_2_, … , *X_N_* as intrinsic variables and reaction rates *k*_1_, *k*_2_, … , *k_M_* as extrinsic variables. Total stochasticity is quantified by the stationary distribution of the intrinsic variables, *p*(**x**). We assume that the invariant probability density of extrinsic variables, *q*(**k**), does not depend on the values of intrinsic variables **x**. Then the law of total probability implies that the stationary probability distribution of intrinsic variables is given by3.5
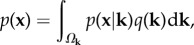


where 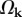 is the parameter space and *p*(**x|k**) represents the invariant density of intrinsic variables conditioned on constant values of kinetic parameters, see the definition below equation (2.1). If distributions *q*(**k**) of extrinsic variables can be determined from high-quality experimental data, then the stationary density can be computed directly by (3.5). If not, the TPA framework enables to test the behaviour of GRNs for different hypothesis about the distribution of the extrinsic variables. The advantage of the TPA is that it efficiently computes the high-dimensional integrals in (3.5) (see electronic supplementary material, appendix S1.4).

### 5.1. Extrinsic noise in FitzHugh–Nagumo model

**Figure 6.**
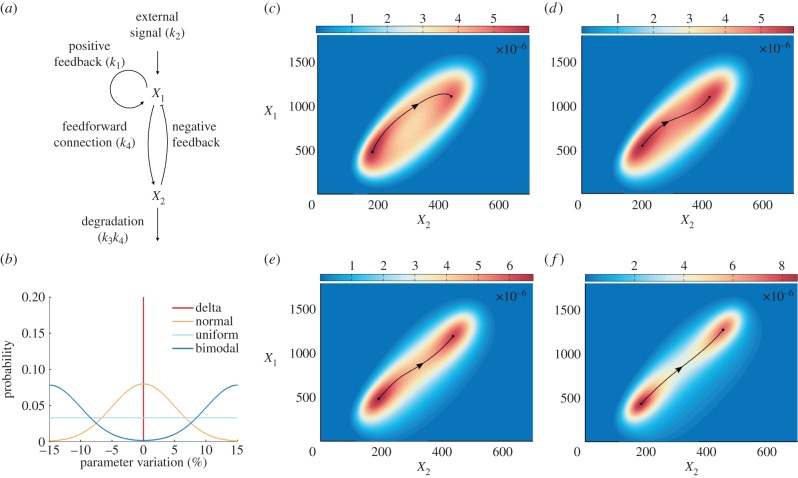
Analysis of the FitzHugh–Nagumo system. (*a*) Schematic of the model. (*b*) Four types of distributions of the extrinsic noise applied to model parameters. (*c*–*f*) Steady-state behaviour of the FitzHugh–Nagumo model with (*c*) constant reaction rates, (*d*) normal, (*e*) uniform and (*f*) bimodal distribution of the model parameters. The tensor-structured computational procedure follows formula (3.6). The directed black curves track the ridges of the stationary distribution and depict the ‘most-likely’ transition paths from the inhibited state to the excited state.

We consider the effect of extrinsic fluctuations on an activator-inhibitor oscillator with simple negative feedback loop: the FitzHugh–Nagumo neuron model which is presented in figure 6*a*. Self-autocatalytic positive feedback loop activates the *X*_1_ molecules, which are further triggered by the external signal. The species *X*_2_ is enhanced by the feed-forward connection and it acts as an inhibitor that turns off the signalling [4]. We perform robustness analysis based on the simulated tensor data in §2.1 (summarized on the third line of table 1). In our computational examples, we assume that 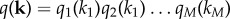, i.e. the invariant distributions of rate constants *k*_1_, *k*_2_, … , *k_M_* are independent. Then (3.5) reads as follows:3.6
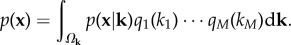

Extrinsic variability in the FitzHugh–Nagumo system is studied in four prototypical cases of *q_i_*, *i* = 1, 2, … , *M*: (i) Dirac delta, (ii) normal, (iii) uniform, and (iv) bimodal distributions, as shown in figure 6*b*. As these distributions have zero mean, the extrinsic noise is not biased. We can then use this information about extrinsic noise to simulate the stationary probability distribution of intrinsic variables by (3.6).

When the extrinsic noise is omitted, the inhibited and excited states are linked by a volcano-shaped oscillatory probability distribution (figure 6*c*). At the inhibited state, *X*_1_ molecules first get activated from the positive feedback loop, and then excite *X*_2_ molecules by feed-forward control. The delay between the excitability of the two molecular species gives rise to the path (solid line) describing switching from the inhibited state to the excited state (figure 6*c*). If the normal or uniform noise are introduced to the extrinsic variables, then the path becomes straighter (figure 6*d*,*e*). This suggests that, once *X*_1_ molecules get excited or inhibited, *X*_2_ molecules require less time to response.

GRNs with stronger negative feedback regulation gain higher potential to reduce the stochasticity. This argument has been both theoretically analysed [45,46], and experimentally tested for a plasmid-borne system [47]. We have shown that the extrinsic noise reduces the delay caused by the feedback loop (figure 6*d*). If we further increase the variability of the extrinsic noise, then the delay caused by the feedback loop is further reduced (figure 6*e*). In the case of the bimodal distribution of extrinsic fluctuations, the most-likely path linking the inhibited and excited states even shrinks into an almost straight line (figure 6*f*). This means that, for the same level of the inhibitor *X*_2_, the number of the activator *X*_1_ is lower, i.e. the presented robustness analysis shows that the behaviour of stochastic GRNs with negative feedback regulation can benefit from the extrinsic noise.

## 6. Discussion

We have presented the TPA of stochastic reaction networks and illustrated that the TPA can (i) calculate and store the parametric steady-state distributions; (ii) infer and analyse stochastic models of GRNs. To explore high-dimensional state space 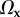 and parameter space 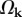, the TPA uses a recently proposed low-parametric tensor-structured data format, as presented in equation (2.2). Tensor methods have been recently used to address the computational intensity of solving the CME [16,48]. In this paper, we have extended these tensor-based approaches from solving the underlying equations to automated parametric analysis of the stochastic reaction networks. One notable advantage of the tensor approach lies in its ability to capture all probabilistic information of stochastic models all over the parameter space into one single tensor-formatted solution, in a way that allows linear scaling of basic operations with respect to the number of dimensions. Consequently, the existing algorithms commonly used in the deterministic framework can be directly used in stochastic models via the TPA. In this way, we can improve our understanding of parameters in stochastic models.

**Figure 7.**
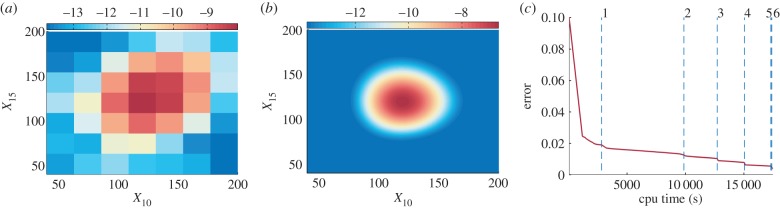
The computation of the stationary distribution using the TPA for a 20-dimensional reaction chain. The CFPE is successively solved on seven grid levels with an increasing number of nodal points. The logarithm of the marginal stationary distribution in the *X*_10_–*X*_15_ plane computed on (*a*) the initial coarsest level; and (*b*) the finest grid level. (*c*) The convergence of the total error versus the computational time. The error is obtained by comparing the marginal distribution of the computed steady-state distribution with the exact solution of the corresponding CME. The vertical dashed lines correspond to the grid levels. The grid size details on each grid level are given in electronic supplementary material, table S8.

To overcome technical (numerical) challenges, we have introduced two main approaches for successful computation of the steady-state distribution. First, we compute it using the CFPE approximation which provides additional flexibility in discretizing the state space 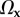. The CFPE admits larger grid sizes for numerical simulations than the unit grid size of the CME. In this way, the resulting discrete operator is better conditioned. We illustrate this using a 20-dimensional problem introduced in the last line of table 1 and in electronic supplementary material, appendix S2.4. To compute the stationary distribution, a multi-level approach is implemented, where the steady-state distribution is first approximated on a coarse grid, and then interpolated to a finer grid as the initial guess (see electronic supplementary material, appendix S1.3, for more details). The results are plotted in figure 7. Second, we introduce the adaptive inverse power iteration scheme tailored to current tensor solvers of linear systems, see electronic supplementary material, appendix S1.3, for technical details. As tensor linear solvers are less robust especially for ill-conditioned problems, it is necessary to carefully adapt the shift value during the inverse power iterations in order to balance the conditioning and sufficient speed of the convergence. We would like to emphasize the importance of these improvements, because the TPA is mainly limited by the efficiency of computing steady-state distributions, rather than by the problem dimension, *N* + *K*. Both the computational efficiency and the separation rank *R* are negatively correlated with the relaxation time of the reaction network. Reaction networks exhibiting bistable or oscillating behaviours usually have larger relaxation times. This explains some counterintuitive results in table 1, namely the smaller memory requirements and shorter computational times of the 20-dimensional reaction chain in comparison with the seven-dimensional cell cycle model. In particular, the TPA can be applied to systems with dimensionality *N* + *K* greater than 20, provided that they have small relaxation times.

Techniques for the parameter inference and bifurcation analysis of stochastic models have been less studied in the literature than the corresponding methods for the ODE models. One of the reasons for this is that the solution of the CME is more difficult to obtain than solutions of mean-field ODEs. This has been partially solved by the widely used Monte Carlo methods, such as the Gillespie SSA, which can be used to estimate the required quantities [29]. Advantages of Monte Carlo methods are especially their relative simplicity and easy parallelization. The TPA provides an alternative approach. The TPA uses more complex data structures and algorithms than the Gillespie SSA, but it enables to compute the whole probability distribution for all combinations of parameter values at once. The TPA stores this information in the tensor format. If the state and parameter spaces have a higher number of dimensions, then the Monte Carlo methods would have problems with storing computed stationary distributions. Another advantage of the TPA is that it produces smooth data, see e.g. figure 3 for the data over the parameter space and figure 5 for the data in the state space. This is important for a stable convergence in the gradient-based optimization algorithms [49] and for reliable analysis of stochastic bifurcations. Monte Carlo methods provide necessarily noisy and hence non-smooth data that may cause problems for these methods.

Parameter inference of stochastic models can make use of various statistical measures, such as the variance and correlations. Monte Carlo approaches are widely used to compute these quantities, but they may be computationally expensive. The TPA provides an alternative approach. Once we compute the stationary distribution for the desired ranges of parameter values and store it in the tensor format, we can use the tensor operation techniques (see electronic supplementary material, appendix S1.4) to efficiently compute many different statistical measures from the same stationary distribution. If the results of the used statistical measure and chosen method are not satisfactory, we can modify or completely change both and try to infer the parameters again. As the stationary distribution is stored, the modifications and changes can be done with low computational load. Namely, no stochastic simulations are needed. In addition, as the stationary distribution contains complete information about the stochastic steady state, it can be used to compute practically any quantity for comparison with experimental data. We have illustrated several different parametric studies in figures 1*b*, 2 and 3. All these results are based on a single tensor solution presented in §2.1 (table 1).

We would like to note that the presented inference is based solely on the steady-state distributions, and not on the time-dependent trajectories. Consequently, parameter estimation of the Schlögl system needs to be performed with at least one model parameter fixed at its true value. Nevertheless, the time evolution can be incorporated into the TPA framework. We can consider the time *t* as an additional dimension in the tensor data [50], i.e. we can compute 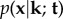, where 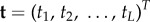 is a vector of temporal samples. Adding a temporal dimension to the separated tensor data increases the storage requirements and computational complexity from order *O*(*N* + *K*) to order *O*(*N* + *K* + 1). Then, the existing trajectory-based inference methods [51] can be applied to the computed tensor data 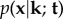. Let us also note that it is relatively straightforward to use the TPA framework to study the parameter sensitivity of stochastic systems (i.e. to quantify the dependence of certain quantities of interest on continuous changes in model parameters). A systematic way for conducting the sensitivity analysis is illustrated in electronic supplementary material, appendix S1.5, using the fission yeast cell cycle model.

## Supplementary Material

Supporting Information Appendix
