# Supplementary material for: Tensor methods for parameter estimation and bifurcation analysis of stochastic reaction networks
Source: J R Soc Interface. 2015 Jul 6;12(108):20150233. doi: 10.1098/rsif.2015.0233 (PMC4528587; doi:10.1098/rsif.2015.0233)
Supplement: Supporting Information Appendix [file rsif20150233supp1.pdf]

Supporting Information Appendix for:

Tensor methods for parameter estimation and bifurcation analysis of stochastic reaction networks

Shuohao Liao*, Tomáš Vejchodský†, Radek Erban*

S1 Methods

All models studied by the TPA are given in terms of well-mixed chemical systems where the system state changes according to the chemical reactions (2.1) (here, equation label (2.1) refers to the corresponding equation in the paper). Probability that a reaction occurs is determined by the propensity function

$$\alpha_j(\mathbf{x}, k_j) = k_j\, \tilde{\alpha}_j(\mathbf{x}), \quad j = 1, 2, \ldots, M, \tag{S1}$$

with the non-parametric part $\tilde{\alpha}_j(\mathbf{x})$ given by

$$\tilde{\alpha}_j(\mathbf{x}) = \exp\left[\left(1 - \sum_{i=1}^{N} \nu_{j,i}^{-}\right) \log V\right] \prod_{i=1}^{N} (\nu_{j,i}^{-})! \binom{x_i}{\nu_{j,i}^{-}},$$

where V is the volume of the reactor. The stationary distribution $p\big(\mathbf{x} \,|\, \mathbf{k}\big)$, where $\mathbf{k} = (k_1, k_2, \ldots, k_K)^T$ is the subset of kinetic rate constants for which the parameter analysis is considered, can be computed as the exact solution of the chemical master equation (CME) [52]. However, for the computational reasons, we will approximate it by the solution of the stationary chemical Fokker-Planck equation (CFPE) [18], which can be written as

$$\mathcal{A}(\mathbf{x}, \mathbf{k})\, p\big(\mathbf{x} \,|\, \mathbf{k}\big) = 0,$$

where

$$\begin{aligned} \mathcal{A}(\mathbf{x}, \mathbf{k})\, p\big(\mathbf{x} \,|\, \mathbf{k}\big) &= -\sum_{i=1}^{N} \frac{\partial}{\partial x_i} \left(\sum_{j=1}^{M} \nu_{j,i}\, \alpha_j(\mathbf{x}, k_j)\, p\big(\mathbf{x} \,|\, \mathbf{k}\big) \right) \\ &+ \frac{1}{2} \sum_{i,i'=1}^{N} \frac{\partial^2}{\partial x_i \partial x_{i'}} \left(\sum_{j=1}^{M} \nu_{j,i}\, \nu_{j,i'}\, \alpha_j(\mathbf{x}, k_j)\, p\big(\mathbf{x} \,|\, \mathbf{k}\big) \right), \end{aligned} \tag{S2}$$

is the parametric Fokker-Planck operator. We use the tensor structures to compute $p\big(\mathbf{x} \,|\, \mathbf{k}\big)$ simultaneously for ranges of values of reaction rates $\mathbf{k}$. To achieve this, we split the model parameters from the state variables in a multiplicative way. Considering the definition of propensity functions (S1), we can split the parametric Fokker-Planck operator (S2) into M terms as

$$\mathcal{A}(\mathbf{x}, \mathbf{k}) = k_1 \mathcal{A}^{[1]}(\mathbf{x}) + \cdots + k_M \mathcal{A}^{[M]}(\mathbf{x}), \tag{S3}$$

where the non-parametric operator $\mathcal{A}^{[j]}(\mathbf{x})$ describes the normalised transition properties of the j-th reaction, and is defined (for any twice differentiable function f) by

$$\mathcal{A}^{[j]}(\mathbf{x})\, f(\mathbf{x}) = -\sum_{i=1}^{N} \nu_{j,i} \frac{\partial}{\partial x_i} \Big(\tilde{\alpha}_j(\mathbf{x})\, f(\mathbf{x}) \Big) + \frac{1}{2} \sum_{i,i'=1}^{N} \nu_{j,i'}\, \nu_{j,i} \frac{\partial^2}{\partial x_i \partial x_{i'}} \Big(\tilde{\alpha}_j(\mathbf{x})\, f(\mathbf{x}) \Big). \tag{S4}$$

*Mathematical Institute, University of Oxford, Radcliffe Observatory Quarter, Woodstock Road, Oxford OX2 6GG, United Kingdom; e-mails: liao@maths.ox.ac.uk; erban@maths.ox.ac.uk

†Institute of Mathematics, Czech Academy of Sciences, Zitna 25, 115 67 Praha 1, Czech Republic; e-mail vejchod@math.cas.cz

Let us note that the definition of propensity functions (S1) relies on the law of mass action. However, the TPA methodology is applicable even for more general definitions [53]. If the propensity functions depend nonlinearly on the kinetic rates, as in [54] for example, then the TPA methodology can be used provided a multiplicative splitting (S3) of the parametric Fokker-Planck operator is possible. Such splitting is always possible if the propensities can be written as a product of two terms, where the first term depends only on kinetic rates, and the second term on state variables.

The splitting of the parametric Fokker-Planck operator (S3) implies a perfect collinear relationship between the kinetic rate parameters. In the context of parameter estimation this means that a single constraint (like mean and variance) restricts the original K-dimensional parameter space to an $(K-1)$-dimensional subspace of parameter values that comply with the constraint. Therefore, if a direct comparison between a model and a sample is not possible, a necessary condition to statistically infer $K \leq M$ parameters of a stochastic system is to define at least K constraints.

S1.1 Tensorization

We consider the state variable $\mathbf{x}$ in a bounded domain $\Omega_{\mathbf{x}} \subset (0,\infty)^N$. Similarly, the kinetic rates $\mathbf{k}$, which are varied during the parametric analysis, are considered in $\Omega_{\mathbf{k}} \subset [0,\infty)^K$ where $K \leq M$. In order to utilize the tensor structures, we assume that $\Omega_{\mathbf{x}} = \mathcal{I}_1 \times \cdots \times \mathcal{I}_N$ and $\Omega_{\mathbf{k}} = \mathcal{J}_1 \times \cdots \times \mathcal{J}_K$, where $\mathcal{I}_d = (a_d^x, b_d^x)$, $d = 1,2,\ldots,N$, are open intervals and $\mathcal{J}_\ell = [a_\ell^k, b_\ell^k]$, $l = 1,2,\ldots,K$, are closed intervals.

We consider homogeneous Dirichlet boundary conditions on the boundary of $\Omega_{\mathbf{x}}$. We approximate the stationary distribution by the (normalized) eigenfunction of $\mathcal{A}(\mathbf{x},\mathbf{k})$ corresponding to the eigenvalue closest to zero. Since the Fokker-Planck operator $\mathcal{A}(\mathbf{x},\mathbf{k})$ is an elliptic operator, the largest eigenvalue converges to 0 from below as the size of $\Omega_{\mathbf{x}}$ increases to infinity. In particular, if we choose a sufficiently large computational domain $\Omega_{\mathbf{x}}$, then the largest eigenvalue will be close to zero and the Dirichlet boundary conditions will not cause any substantial error.

The chemical Fokker-Planck operator (S4) is discretized in $\Omega_{\mathbf{x}}$ by the finite difference method. We consider tensor grids [55] in both $\Omega_{\mathbf{x}}$ and $\Omega_{\mathbf{k}}$. The tensor grid in $\Omega_{\mathbf{x}}$ has nodes $(x_{1,i_1},\ldots,x_{N,i_N})$, $i_d = 1,2,\ldots,n_d$, $d = 1,2,\ldots,N$. There are n_d points $x_{d,i_d} = a_d^x + i_d h_d^x$, $i_d = 1,2,\ldots,n_d$, in every $\mathcal{I}_d$ with the grid size $h_d^x = (b_d^x - a_d^x)/(n_d+1)$, $d = 1,2,\ldots,N$. Similarly, we define tensor grid $(k_{1,j_1},\ldots,k_{K,j_K})$ in $\Omega_{\mathbf{k}}$, where $k_{\ell,j_\ell} = a_\ell^k + (j_\ell - 1)h_\ell^k$, $j_\ell = 1,2,\ldots,m_\ell$, form a uniform partition of $\mathcal{J}_\ell$ with the grid size $h_\ell^k = (b_\ell^k - a_\ell^k)/(m_\ell - 1)$, $\ell = 1,2,\ldots,K$. Note that the boundary points a_d^x and b_d^x, $d = 1,2,\ldots,N$, are not present in the tensor grid due to the Dirichlet boundary conditions.

The values of the stationary distribution $p(\mathbf{x}\,|\,\mathbf{k})$ at the nodal points are organized as an $(N+K)$-dimensional tensor $\bar{\mathbf{p}} \in \mathbb{R}^{n_1\times\cdots\times n_N\times m_1\times\cdots\times m_K}$ with entries

$$\bar{\mathbf{p}}_{i_1,\ldots,i_N,j_1\ldots,j_K} = p(x_{1,i_1},\ldots,x_{N,i_N}|k_{1,j_1},\ldots,k_{K,j_K}). \tag{S5}$$

In the traditional matrix-vector approach, we would organize the entries of $\bar{\mathbf{p}}$ into a long vector. However, the tensor structure is more natural, because it corresponds to the original physical position of the nodes within the state and parameter space [56]. Finally, let us note that if $n = n_1 = \cdots = n_N$ and $m = m_1 = \cdots = m_K$ then there is $n^N m^K$ entries in the tensor $\bar{\mathbf{p}}$. Thus, the number of memory places to store the tensor $\bar{\mathbf{p}}$ grows exponentially with N and K. In the next subsection, we present the main idea of the separated representation of tensors that allows to solve this problem.

S1.2 Separation of dimensions

The main idea of the separated (or low-parametric) representation is to approximate tensor $\bar{\mathbf{p}}$ by the following sum of rank-one tensors:

$$\bar{\mathbf{p}} \approx \sum_{r=1}^{R} \underbrace{\phi_1^{[r]} \otimes \cdots \otimes \phi_N^{[r]}}_{\text{state space}} \otimes \underbrace{\psi_1^{[r]} \otimes \cdots \otimes \psi_K^{[r]}}_{\text{parameter space}}, \tag{S6}$$

where $\phi_d^{[r]} \in \mathbb{R}^{n_d}$, $d = 1, 2, \ldots, N$, and $\psi_\ell^{[r]} \in \mathbb{R}^{m_\ell}$, $\ell = 1, 2, \ldots, K$, are factor vectors, R is known as the separation rank, and symbol $\otimes$ denotes the tensor product of vectors [57]. Let us recall that the tensor product $\mathbf{v}_1 \otimes \mathbf{v}_2 \otimes \cdots \otimes \mathbf{v}_N$ of vectors $\mathbf{v}_d \in \mathbb{R}^{n_d}$, $d = 1, 2, \ldots, N$, is defined as a tensor $\bar{\mathbf{v}} \in \mathbb{R}^{n_1 \times n_2 \times \cdots \times n_N}$ with entries $v_{i_1, i_2, \ldots, i_N} = v_{1,i_1} v_{2,i_2} \cdots v_{N,i_N}$.

Representation (S6) has the potential to solve high-dimensional problems. Indeed, if we consider for simplicity $n = n_1 = \cdots = n_N$ and $m = m_1 = \cdots = m_K$ then the representation (S6) requires to store $(nN + mK)R$ numbers only. For moderate values of R this is substantially less than the number of entries of $\bar{\mathbf{p}}$. Moreover, low-parametric representations such as (S6) enable to perform algebraic operations in an efficient way, see Section S1.4.

The accuracy of the separated representation (S6) depends on the choice of the factor vectors and on the size of the tensor rank R. Clearly, the higher rank enables higher accuracy, but requires higher computational and storage costs. In practical computations, the rank R is dynamically controlled using algorithms for tensor truncation, see Section S1.3. Let us note that the representation (S6) is known as the canonical polyadic decomposition [58]. However, due to reasons connected with the stability of the tensor truncation algorithms, it is not suitable for actual computation and more stable tensor formats have to be employed [22]. We have introduced the canonical polyadic decomposition (S6) due to its simplicity to illustrate the main idea of the separate representation of tensors.

For certain simple problems, like birth-death process, the separable representation of the stationary distribution can be derived explicitly. However, in general, we have to compute the stationary distribution in the form (S6). To achieve this, we need to express the discretized Fokker-Planck operator in a separable form as well. Based on the structure of $\mathcal{A}(\mathbf{x}|\mathbf{k})$ in (S3), the discretization of the parametric Fokker-Planck operator can be divided into two steps: decomposing the non-parametric part (see Section S1.2.1) and the parametric part (see Section S1.2.2).

S1.2.1 Decomposition of the non-parametric part

We use the finite differences to discretize the derivatives in the non-parametric operators $\mathcal{A}^{[j]}(\mathbf{x})$ in (S4), see e.g [59]. The separated tensor representation does not require high-dimensional difference stencils. Instead, just one-dimensional differences are needed. Further, since the standard finite difference discretizations of differential operators yield matrices, we organize their entries naturally into tensors. In this situation we speak about tensor matrices and denote them in capital bold font. The idea is exactly the same as in (S5), where we organized a long vector into a tensor.

Thus, the finite difference matrix approximating the non-parametric operator $\mathcal{A}^{[j]}(\mathbf{x})$ in (S4) can be expressed as the following tensor matrix:

$$\mathbf{A}^{[j]} = -\sum_{i=1}^{N} \nu_{j,i} \mathbf{G}^{[i;j]} + \frac{1}{2} \sum_{i,i'=1}^{N} \nu_{j,i} \nu_{j,i'} \mathbf{F}^{[i,i';j]}, \quad j = 1, 2, \ldots, M, \tag{S7}$$

where tensor matrices $\mathbf{G}^{[i;j]}$ and $\mathbf{F}^{[i,i';j]}$ refer to tensor-structured discretizations of the summands in the first and second sums in (S4), respectively, and are determined by

$$\begin{aligned}
\mathbf{G}^{[i;j]} &= \tilde{v}_j \, H_1^{[j]} \otimes \cdots \otimes D_i H_i^{[j]} \otimes \cdots \otimes H_N^{[j]}, \\
\mathbf{F}^{[i,i';j]} &= \tilde{v}_j \, H_1^{[j]} \otimes \cdots \otimes D_i H_i^{[j]} \otimes \cdots \otimes D_{i'} H_{i'}^{[j]} \otimes \cdots \otimes H_N^{[j]}, \qquad \text{for} \quad i < i', \\
\mathbf{F}^{[i,i;j]} &= \tilde{v}_j \, H_1^{[j]} \otimes \cdots \otimes D_i D_i H_i^{[j]} \otimes \cdots \otimes H_N^{[j]},
\end{aligned}$$

where the volume scaling coefficient is $\tilde{v}_j = \exp\left[(1 - \sum_{i=1}^{N} \nu_{j,i}^{-}) \log V\right]$. Here, $H_i^{[j]} \in \mathbb{R}^{n_i \times n_i}$ and $D_i \in \mathbb{R}^{n_i \times n_i}$ for $i = 1, 2, \ldots, N$ and $j = 1, 2, \ldots, M$ are matrices and, thus, the tensor product $\otimes$ works in the same way as the Kronecker product. Matrix D_i is the central difference matrix with entries $-1/(2h_i^x)$ and $1/(2h_i^x)$ distributed along its super- and sub-diagonal, respectively. Matrix $H_i^{[j]}$ is diagonal with diagonal entries

$$H_i^{[j]}(\ell, \ell) = (\nu_{j,i}^{-})! \binom{x_{i,\ell}}{\nu_{j,i}^{-}} \qquad \text{for} \quad \ell = 1, 2, \ldots, n_i.$$

We observe that tensor matrices $\mathbf{G}^{[i;j]}$ and $\mathbf{F}^{[i,i';j]}$ are expressed in a separated representation similar to (S6) with the separation rank $R = 1$. Consequently, the non-parametric operator $\mathbf{A}^{[j]}$ in (S7) admits separable representation of rank $R = N(N+1)/2 + N = N^2/2 + 3N/2$. Thus, any further algebraic operation on $\mathbf{A}^{[j]}$ would contribute to the overall complexity growing quadratically in terms of number of chemical species.

S1.2.2 Decomposition of the parametric part

Having the low-parametric discrete tensor-structured representations (S7) of the non-parametric operators $\mathbf{A}^{[j]}$, we write a discrete tensor-structured representation of the parametric Fokker-Planck operator (S3) as

$$\mathbf{A} = \mathbf{A}^{[1]} \otimes K_1 \otimes I_2 \otimes \cdots \otimes I_M + \mathbf{A}^{[2]} \otimes I_1 \otimes K_2 \otimes \cdots \otimes I_M + \cdots + \mathbf{A}^{[M]} \otimes I_1 \otimes I_2 \otimes \cdots \otimes K_M, \quad \text{(S8)}$$

where $K_j \in \mathbb{R}^{m_j \times m_j}$ denotes a diagonal matrix whose diagonal entries correspond to the grid nodes of the j-th parameter, i.e., $K_j(\ell, \ell) = k_{j,\ell}$ for $\ell = 1, 2, \ldots, m_j$ and $j = 1, 2, \ldots, M$.

Equation (S8) is a low-parametric tensor representation of the discretized parametric Fokker-Planck operator with separation rank $M(N^2/2 + 3N/2)$. This rank grows linearly with the number of chemical reactions M and quadratically with the number of chemical species N. Then, the parametric steady state distribution of the form (S6) is solved as the eigenvector of $\mathbf{A}$ corresponding to the eigenvalue closest to zero (see Section S1.3).

S1.3 Solving the stationary CFPE in tensor format

Let $\mathbf{A}$ be the tensor-structured parametric Fokker-Planck operator assembled in (S8). Our goal is to approximate the stationary distribution by the eigenvector $\bar{\mathbf{p}}$ corresponding to the eigenvalue λ_{min} which is closest to zero, i.e.

$$\mathbf{A}\bar{\mathbf{p}} = \lambda_{\text{min}}\bar{\mathbf{p}}. \quad \text{(S9)}$$

A standard method to find the required eigenpair of $\mathbf{A}$ is the inverse power method, and here we modify the original algorithm for better implementations in tensor-structured computations.

Adaptive inverse power algorithm. The main building block is the fact that, beginning with an initial guess $\bar{\mathbf{p}}_0$ and given a shift value σ, the inverse power scheme,

$$(\mathbf{A} - \sigma\mathbf{I})\bar{\mathbf{p}}_{k+1} = \frac{\bar{\mathbf{p}}_k}{\|\bar{\mathbf{p}}_k\|}, \quad k = 0, 1, \ldots \quad \text{(S10)}$$

would converge to the eigenvector corresponding to the eigenvalue closest to the chosen shift σ, provided that the eigenvalue is of multiplicity one. Since all eigenvalues of the Fokker-Planck operator have negative real parts, we choose $\sigma \geq 0$. We do it adaptively based on the performance of the tensor linear solver, i.e. $\sigma \equiv \sigma_k$ in (S10).

We apply the alternating minimum energy method (AMEN) [60] to solve the linear system (S10). Given an initial N-dimensional tensor $\bar{\mathbf{p}}$, the AMEN method minimises the residual in a single dimension at a time with other dimensions fixed, and alternates the dimensions from 1 to N. The entire *sweep* repeats until a convergence criterion is satisfied. Typically, smaller shift σ makes the whole inverse power method converge faster to the steady state solution, however, within each inverse iteration (S10), the AMEN may require many sweeps to achieve a reasonable tolerance. Thus, our strategy is to double the shift value σ when the solver reach certain upper threshold, and half σ to seek for better convergence for the whole procedure when the AMEN converges with only a few sweeps.

Another extension arises from a feature of tensor-structured data format. The tensor separation rank R can increase rapidly over successive algebraic operations, making the representation untenable. To avoid uncontrollable growth of the separation rank throughout the computation, we need to reduce it by adaptively changing the involving factor vectors while maintaining the required accuracy. This procedure is usually called tensor truncation:

$$\bar{\mathbf{p}}^* = \Gamma(\bar{\mathbf{p}}), \quad \text{(S11)}$$

where operator Γ is the truncation operator, and $\operatorname{rank}(\bar{\mathbf{p}}^*) < \operatorname{rank}(\bar{\mathbf{p}})$. Although finding the optimal tensor separation rank is still an open question of the ongoing research, the tensor train format, together with its SVD-based tensor truncation algorithm, is a stable and useful prototype for our implementations, and we refer the readers to [22] for further details.

Consequently, the adaptive inverse power method used in the TPA is summarised as follows:

Step 0. Initialize: initial guess $\bar{\mathbf{p}}_0$; shift value $\sigma = \sigma_0$; stopping criterion ε; maximum number of AMEN sweeps in each inverse iteration N_{max}; thresholds to increase (N_{in}) and decrease (N_{de}) the shift value.

Step 1. Solve the k-th tensor-structured inverse iteration (S10) up to N_{max} sweeps.

Step 2. Check the number of sweep N_{comp} for the AMEN solver to converge:

- **2a.** If $N_{comp} > N_{in}$, let $\sigma = 2\sigma$ and jump back to Step 1.
- **2b.** If $N_{de} < N_{comp} \leq N_{in}$, go to Step 3.
- **2c.** If $N_{comp} \leq N_{de}$, $\sigma = \sigma/2$ and go to Step 3.

Step 3. Truncate the tensor separation rank as in (S11).

Step 4. Check the stopping criterion:

- **4a.** If $\|\bar{\mathbf{p}}_{k+1} - \bar{\mathbf{p}}_k\| > \varepsilon$, let $\bar{\mathbf{p}}_{k+1} = \bar{\mathbf{p}}_k$ and $k = k + 1$, and jump to Step 1.
- **4b.** If $\|\bar{\mathbf{p}}_{k+1} - \bar{\mathbf{p}}_k\| \leq \varepsilon$, return $\bar{\mathbf{p}}_{k+1}$ and exit.

Multi-level acceleration. When the dimensionality of the problem is large, the adaptive scheme discussed above may converge slowly, because on a fixed grid size, the AMEN requires very large shift value σ to solve (S10). Thus, the TPA makes use of a multi-level scheme to accelerate the solution process for high-dimensional problems. The system (S9) is first solved on a coarse grid with grid size $2h$. The approximated stationary solution is then interpolated to a fine grid with grid size h and used as an initial guess. The method continues to solve the system on finer grids until some convergence criteria are achieved.

A key step in the multi-level approach is the interpolation, or prolongation, matrix that transfers the solution on a coarse grid to a fine grid. The prolongation operator has a rank-one tensor structure. Let N-dimensional tensor $\bar{\mathbf{p}} \in \mathbb{R}^{n_1 \times n_2 \times \cdots \times n_N}$ contain the function values on an N-dimensional tensor grid with n_k, $k = 1, 2, \ldots, N$, grid points along each direction. The prolongation operator $\mathbf{P}^{[k]}$ to the k-th dimension is then defined as

$$\mathbf{P}^{[k]} = I \otimes \cdots \otimes I \otimes \underbrace{P_{n_k}^{2n_k}}_{k\text{-th mode}} \otimes I \otimes \cdots \otimes I, \tag{S12}$$

where $P_{n_k}^{2n_k} \in \mathbb{R}^{2n_k \times n_k}$ is the one-dimensional interpolation matrix defined by

$$P_{n_k}^{2n_k} = \frac{1}{2} \begin{pmatrix} 2 & & & \\ 1 & 1 & & \\ & 2 & & \\ & 1 & 1 & \\ & & & \ddots \end{pmatrix}.$$

If tensor $\bar{\mathbf{p}}$ has the rank-R separated representation as (S6) with $n = n_1 = n_2 = \cdots = n_N$, the complexity to interpolate a single dimension is $\mathcal{O}(n)$, and the total complexity of a full interpolation over N-dimensional tensor grid is $\mathcal{O}(nN)$. We summarise the multi-level accelerated adaptive inverse power method as follows:

Step 0. Initialize: initial grid size on the coarsest grid h_1; initial guess $\bar{\mathbf{p}}_0^{(1)}$; initial error tolerance $\varepsilon^{(1)}$; maximum number of grid levels L_{max}; and let $\ell = 1$.

Step 1. Solve the eigenvalue problem (S9) on the ℓ-th level with initial guess $\bar{\mathbf{p}}_0^{(\ell)}$, using the adaptive inverse power method. Return the solution $\bar{\mathbf{p}}_k^{(\ell)}$ that satisfies the error tolerance $\|\bar{\mathbf{p}}_k^{(\ell)} - \bar{\mathbf{p}}_{k-1}^{(\ell)}\| \leq \varepsilon^{(\ell)}$.

Step 2. If $\ell < L_{max}$, interpolate the solution $\bar{\mathbf{p}}_k^{(\ell)}$ to a finer grid by successive application of the prolongation operator $\mathbf{P}^{[k]}$ in (S12) to each dimension. Let $\bar{\mathbf{p}}_0^{(\ell+1)} = \bar{\mathbf{p}}_k^{(\ell)}$, $\varepsilon^{(\ell+1)} = \varepsilon^{(\ell)}/2$, $\ell = \ell + 1$. Go to Step 1.

Step 3. If $\ell = L_{max}$, return the solution $\bar{\mathbf{p}}_k^{(\ell)}$ and exit.

Multi-level approach is used in Section S2.4 to analyse the 20-dimensional chemical system (S17). CPU times for each grid size are shown in Table S8. In general, the operators of both the CME and CFPE are non-symmetric and ill-conditioned, and challenging to handle using tensor-structured solvers [61,62]. Although it can be improved by shortening the time-step [48], the CFPE has a distinctive advantage over the CME for its flexibility in choosing the grid size, which enables to control the accuracy and use of acceleration strategies, such as the presented multi-level approach.

Implementation. The TPA, implemented in MATLAB, is included in the Stochastic Bifurcation Analyzer toolbox available at `http://www.stobifan.org`. The source code relies on the Tensor Train Toolbox [22]. Simulations are performed on a 64-bit Linux desktop equipped with Quad-Core AMD Opteron™ Processor 8356 × 16 and 63 GB RAM.

S1.4 Elementary tensor operations

The computation of the tensor-structured parametric solution $\bar{\mathbf{p}}$ has been described in Section S1.3. In this section, we discuss computational details of post-processing the solution in the form (S6) for parametric analysis. This analysis is based on high-dimensional integration, implemented using the k-mode product described below.

Tensor multiplication: the k-mode product [63]. Let $\bar{\mathbf{p}} \in \mathbb{R}^{n_1 \times n_2 \times \cdots \times n_N}$ be an N-dimensional tensor, the k-mode product of $\bar{\mathbf{p}}$ with a vector $\mathbf{q} \in \mathbb{R}^{n_k}$ is denoted by $\bar{\mathbf{p}} \times_k \mathbf{q}$ and is a tensor of size $n_1 \times n_{k-1} \times 1 \times n_{k+1} \times \cdots \times n_N$. Elementwise, we have

$$(\bar{\mathbf{p}} \times_k \mathbf{q})_{i_1,\ldots,i_{k-1},1,i_{k+1},\ldots,i_N} = \sum_{j_k=1}^{n_k} \bar{\mathbf{p}}_{i_1,\ldots,i_{k-1},j_k,i_{k+1},\ldots,i_N}\, q_{j_k}. \tag{S13}$$

Further, if $\bar{\mathbf{p}}$ can be written as a rank-R tensor, i.e., $\bar{\mathbf{p}} = \sum_{r=1}^{R} \phi_1^{[r]} \otimes \cdots \otimes \phi_N^{[r]}$, then the k-mode product can be evaluated through R one-dimensional inner products:

$$\bar{\mathbf{p}} \times_k \mathbf{q} = \sum_{r=1}^{R} \phi_1^{[r]} \otimes \cdots \otimes \phi_{k-1}^{[r]} \otimes \langle \phi_k^{[r]}, \mathbf{q} \rangle \otimes \phi_{k+1}^{[r]} \otimes \cdots \otimes \phi_N^{[r]}.$$

The $(i_1, i_2, \ldots, i_N)$-th order moment computation in equation (3.3). For tensor-structured parametric solution in (S6), integral (3.3) can be simultaneously approximated for all parameter sets through successive application of the mode product introduced in (S13) as

$$\mu_{[i_1,\ldots,i_N]}(\mathbf{k}^*) \approx h_1^x h_2^x \cdots h_N^x \left(\bar{\mathbf{p}} \times_1 \mathbf{x}_1^{i_1} \times_2 \mathbf{x}_2^{i_2} \times_3 \cdots \times_N \mathbf{x}_N^{i_N} \right), \tag{S14}$$

where $\mathbf{x}_d^{i_d} = (x_{d,1}^{i_d}, x_{d,1}^{i_d}, \ldots, x_{d,n_d}^{i_d})^T$ and h_d^x is the grid size, defined in Section S1.1. The computational complexity of (S14) is $\mathcal{O}(nNR)$, where $n = \max\{n_1, n_2, \ldots, n_d\}$.

Computation of integral in equation (3.6) in the paper. Given the distributions of parameters $q_j(k_j)$ for $j = 1, 2, \ldots, K$, the integral in equation (3.6) can be efficiently computed using the tensor-structured solution (S6) by

$$\bar{\mathbf{p}}_{\mathbf{x}} = h_1^k h_2^k \ldots h_K^k \left(\bar{\mathbf{p}} \times_{N+1} \mathbf{q}_1 \times_{N+2} \mathbf{q}_2 \times_{N_3} \cdots \times_{N+K} \mathbf{q}_K \right), \tag{S15}$$

where the entries of vectors $\mathbf{q}_j$ for $j = 1, 2, \ldots, K$, represent the values of $q_j(k_j)$ at the discrete node points $k_{j,1}, k_{j,2}, \ldots, k_{j,m_j}$ and $h_1^k, h_2^k, \ldots, h_K^k$ are grid sizes in the parameter space, defined in Section S1.1. If $m = m_1 = m_2 = \cdots = m_K = m$ then the complexity of evaluating the approximation (S15) of the K-dimensional integral (3.6) is $\mathcal{O}(mKR)$, which scales linearly with the separation rank R, the number of parameters K, and the number of grid nodes m along each dimension in the parameter space.

Computing transition probability and oscillation amplitude. In the parameter estimation (Figure 2) and sensitivity analysis (Figure S1), we illustrate the results based on the transition probability and oscillation amplitude that are extracted from tensor-structured parametric solution. For example, the probability that, in steady state distribution, the ℓ-th chemical species stays below a certain threshold $\tilde{x}_\ell$, is estimated as follows. We first integrate out all the other dimensions in the state space, and integrate the ℓ-th dimension up to $\tilde{x}_\ell$, i.e.,

$$p(x_\ell \le \tilde{x}_\ell \,|\, \mathbf{k}) = \int_{a_1^x}^{b_1^x} \cdots \int_{a_{\ell-1}^x}^{b_{\ell-1}^x} \int_{a_\ell^x}^{\tilde{x}_\ell} \int_{a_{\ell+1}^x}^{b_{\ell+1}^x} \cdots \int_{a_N^x}^{b_N^x} p(\mathbf{x} \,|\, \mathbf{k}) \, \mathrm{d}\mathbf{x}.$$

In tensor structure, we use N-mode products to compute $p(x_\ell \le \tilde{x}_\ell \,|\, \mathbf{k})$ simultaneously for all parameter combinations by

$$h_1^x h_2^x \cdots h_N^x \left(\bar{\mathbf{p}} \times_1 \mathbf{1} \times_2 \mathbf{1} \times_3 \cdots \times_{\ell-1} \mathbf{1} \times_\ell \mathbf{1}_{\tilde{x}_\ell} \times_{\ell+1} \mathbf{1} \times_{\ell+2} \cdots \times_N \mathbf{1} \right),$$

where $\mathbf{1}$ denotes a vector of all ones. Entries of $\mathbf{1}_{\tilde{x}_\ell}$ are equal to 1 if the corresponding grid point is smaller or equal to $\tilde{x}_\ell$, while its other entries are zero.

S1.5 Sensitivity analysis

The sensitivity indicator for an observable quantity Θ with respect to a parameter k is often computed as a finite difference [64]

$$S(\Theta) \approx \frac{\|\Theta(k + \Delta k) - \Theta(k)\|}{\Delta k} \frac{k}{\|\Theta(k)\|}, \tag{S16}$$

where $\|\cdot\|$ represent a suitable norm, and Δk is a change in the value of k. The model is sensitive to the parameter, if a small Δk yields a large value of $S(\Theta)$. For deterministic models, the observable Θ is usually the steady-state mean concentration. In stochastic setting, we have more options.

For example, let us consider the cell cycle model described in Figure 4(a). We will study the sensitivity with respect to the parameter k_1 for the following three observables Θ: mean concentration of the MPF (Θ_m), the oscillation amplitude (Θ_a) and the steady state distribution (Θ_p). In the case of the oscillation amplitude, we quantify Θ_a as the probability that the molecular population of the active MPF exceeds 400.

In the TPA framework, Θ_m and Θ_a are evaluated for all considered values of k_1 with computational cost scaling linearly with N. More importantly, the tensor-structured data enable direct comparison of two steady state probabilities in the 6-dimensional state space. Namely, the norm $\|\Theta_p(k_1 + \Delta k_1) - \Theta_p(k_1)\|$, needed in (S16), can be directly computed. The results are plotted in Figure S1. We observe that, within the considered range of k_1 (see Table S5), the sensitivity in the steady state distribution (blue curve) dominates in magnitude over $S(\Theta_m)$ and $S(\Theta_a)$. The steady state distribution contains a global information about the system and is more sensitive to parameter changes than derived quantities, like Θ_m and Θ_a.

S2 Description of models used in the illustrative TPA computations

S2.1 Schlögl model

The Schlögl system is defined by chemical reactions listed in Table S1. This table also shows the true values of parameters k_1, k_2, k_3 and k_4. Using this system, we illustrate the capabilities of the TPA for the parameter estimation and identifiablity. Table S2 provides the values of the first three statistical moments and the corresponding weights. The moments have been computed from a time series obtained by a long-time stochastic simulation with the values of parameters given in Table S1. A short segment of the time series is illustrated in Figure 1(a). We then assume that the values of parameters k_1, k_2, k_3 and k_4 are unknown. The TPA enables to evaluate the moment matching distance function given in the paper in equation (3.1), for all values of parameters within the parameter space given in Table S3. The resulting data are stored in the tensor format which enables efficient manipulations and post-processing.

Having the values of the distance function stored in the tensor format, we can easily and quickly obtain further pieces of information. For example, we can find those parameter values which do not fit the moments exactly, but with certain accuracy. More precisely, we consider tolerance $J_{\mathrm{TOL}} = 0.25\,\%$ and visualize in Figures 2(a)–(d) parameter values with distance function J less than J_{TOL}. Alternatively, if the values of moments are not available, we can utilize the TPA for different experimental data – see Figure 1(b) and equation (3.4) in the paper.

Note that all four values of parameters k_1, k_2, k_3 and k_4 cannot be estimated solely from the steady state distribution, because it does not inform us how fast the system reaches the steady state. In particular, if $\{k_i\}_{i=1,2,3,4}$ fit the pseudo-experimental data, then $\{Ck_i\}_{i=1,2,3,4}$ for any $C > 0$ fit these data as well. Therefore, in Figures 1(b) and 2, we fix one of the parameters at its true value and estimate values of the other three.

Table S1: *Reactions and parameters of the Schlögl model.*

Index	Reaction	Kinetic rate[a]	True value
1	$3X \to 2X$	k_1/V^2	$k_1 = 2.5 \times 10^{-4}$
2	$2X \to 3X$	k_2/V	$k_2 = 0.18$
3	$\emptyset \to X$	$k_3\,V$	$k_3 = 2250$
4	$X \to \emptyset$	k_4	$k_4 = 37.5$

[a]The reacting volume is set to $V = 1$ unit.

Table S2: *Moments estimated from stochastic simulation of the Schlögl model.*

Moment order	Value	Weight
1	$\hat{\mu}_1 = 261.32$	$\beta_1 = 1$
2	$\hat{\mu}_2 = 2.03 \times 10^4$	$\beta_2 = 100$
3	$\hat{\mu}_3 = -2.04 \times 10^5$	$\beta_3 = 0.001$

S2.2 Cell cycle model

The cell cycle model consists of six chemical species (see Table S5) which are subject to chemical reactions listed in Table S4, see also Figure 4(a) in the paper. We have used this model to show how the TPA can be used to analyse bifurcations for high-dimensional problems, see Figures 4(b), 4(c) and 5 in the paper. In addition, we have used this system to discuss the sensitivity of various quantities in the stochastic model, see Figure S1 and Section S1.5.

Table S3: *Properties of molecular and rate variables in the Schlögl model.*

Type	Notation	Range	No. of nodes
Species	X	$[0, 1000]$	1024
Rate	k_1	$[2.43 \times 10^{-4}, 2.58 \times 10^{-4}]$	128
Rate	k_2	$[0.17, 0.19]$	128
Rate	k_3	$[2134, 2266]$	128
Rate	k_4	$[36.08, 38.63]$	128

Table S4: *Reactions and parameters of the cell cycle model.*

Index	Reaction	Kinetic rate[a]	Parameter(s)
1	$M \to C_2 + YP$	k_1	bifurcation parameter
2	$\emptyset \to Y$	$k_2\,V$	$k_2 = 0.015$
3	$CP + Y \to pM$	k_3/V	$k_3 = 200$
4	$pM \to M$	$k'_4 + k_4\,(M/V)^2$	$k_4 = 180$, $k'_4 = 0.018$
5	$M \to pM$	$k_5\,tP$	$k_5 = 0$, $tP = 0.001$
6	$Y \to \emptyset$	k_6	$k_6 = 0$
7	$YP \to \emptyset$	k_7	$k_7 = 0.6$
8	$C_2 \to CP$	$k_8\,tP$	$k_8 = 1000$
9	$CP \to C_2$	k_9	$k_9 = 1000$

[a]The volume corresponds to a single cell and is set to $V = 5 \times 10^3$ units.

Table S5: *Properties of molecular and rate variables in the cell cycle model.*

Type	Name	Notation	Range	No. of nodes
Species	cdc2	C2	$[2230, 4990]$	N/A[a]
Species	cdc2-P	CP	$[10, 70]$	256
Species	p-cyclin-cdc2-p	pM	$[0, 1500]$	256
Species	p-cyclin-cdc2	M	$[0, 1200]$	256
Species	cyclin	Y	$[20, 70]$	256
Species	p-cyclin	YP	$[0, 700]$	256
Rate	degradation rate of active MPF	k_1	$[0.25, 0.4]$	64

[a]Discretisation of cdc2 is not applicable here, since this variable is eliminated by the conservation law of cdc2 assumed by the original author.

S2.3 FitzHugh-Nagumo model

The FitzHugh-Nagumo model consists of five chemical reactions between two chemical species. It is illustrated in Figure 6(a) in the paper and the parameter ranges and mean values are provided in Tables S6 and S7. This model is used to show how the TPA can assess the influence of the extrinsic noise, see Figure 6 in the paper.

S2.4 A chemical reaction system in 20 dimensions

We consider a reaction chain of 20 molecular species:

$$\emptyset \xrightarrow{k_0} X_1 \underset{k_{-1}}{\overset{k_1}{\rightleftarrows}} X_2 \underset{k_{-2}}{\overset{k_2}{\rightleftarrows}} \cdots \underset{k_{-18}}{\overset{k_{18}}{\rightleftarrows}} X_{19} \underset{k_{-19}}{\overset{k_{19}}{\rightleftarrows}} X_{20} \xrightarrow{k_{20}} \emptyset, \tag{S17}$$

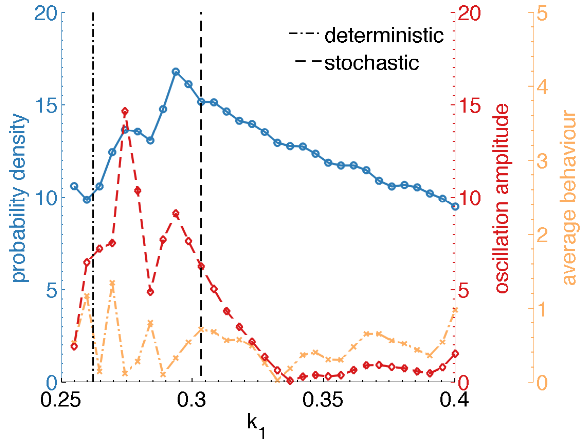

Figure S1: *Sensitivity indicators $S(\Theta)$ calculated by* (S16) *for* 32 *equidistant nodes within the range* $[0.25, 0.4]$ *of parameter k_1 (see Table* S5 *and Section* S1.5*). Three observables are considered: stationary distribution Θ_p (blue), oscillation amplitude Θ_a (red) and average number Θ_m of active MPF (orange). The dot-dashed and dashed lines indicate parameter values for which Figures* 4(b) and 4(c) *in the paper were computed, i.e. $k_1 = 0.2694$ (deterministic bifurcation point) and $k_1 = 0.3032$, respectively.*

Table S6: *Properties of molecular and rate variables in the FitzHugh-Nagumo model.*

Type	Notation	Range	No. of nodes
Species	X_1	$[0, 1800]$	256
Species	X_2	$[0, 700]$	256
Rate	k_1	$[0.17, 0.23]$	128
Rate	k_2	$[0.952, 0.1288]$	128
Rate	k_3	$[2.125, 2.875]$	128
Rate	k_4	$[0.0892, 0.1207]$	128

Table S7: *Reactions and parameters of the FitzHugh-Nagumo model.*

Index	Reaction	Kinetic rate[a]	Mean value
1	$X_1 \to 2X_1$	$(X_1 - k_1\,V)(V - X_1)$	$k_1 = 0.2$
2	$X_1 \to \emptyset$	X_2	N/A
3	$\emptyset \to X_1$	$k_2\,V$	$k_2 = 0.112$
4	$X_2 \to \emptyset$	$k_3\,k_4$	$k_3 = 2.5$
5	$X_1 \to X_1 + X_2$	k_4	$k_4 = 0.105$

[a]The system volume is $V = 2 \times 10^3$ units.

where $k_0 = 12$, $k_i = 0.2$ for $i = 1, 2, \ldots, 20$, and $k_{-j} = 0.1$ for $j = 1, 2, \ldots, 19$. A multi-level approach is implemented to solve the underlying CFPE, where the steady state distribution is first approximated on a coarse grid, and then interpolated to a finer grid (see Section S1.3). The approximate solution on the initial coarsest level is plotted in Figure 7(a), and the solution on the finest grid level is plotted in Figure 7(b). We also note that the CME for the reaction chain, as a monomolecular reaction system, can be solved explicitly. Its steady state distribution is

Table S8: *Multi-level discretisation for the* 20*-dimensional reaction chain* (S17).

Level	1	2	3	4	5	6	7
No. of nodes n	8	16	32	64	128	256	512
Grid size h	20	10	5	2.5	1.25	0.625	0.3125
CPU time† ($\times 10^3$ sec)	2.82	7.02	2.84	2.3	2.25	0.08	0.10

† The computational time that the simulation spent on each grid level. The numbers correspond to the segments of the time axis in Figure 7(c) separated by the dashed lines.

a product Poisson distribution [15]. This means that the marginal probability distribution of the i-th species is

$$p_i(x_i) = \frac{\phi_i^{x_i}}{x_i!} \exp(-\phi_i), \qquad\qquad i = 1, 2, \ldots, 20,$$

where ϕ_i is the corresponding steady state solution of the i-th species in the deterministic reaction rate equation. Thus, we can evaluate the accuracy of the computed steady state distribution in the tensor format by computing its marginal distribution, interpolating to the integer states, and comparing with the exact solution. The error convergence is plotted in Figure 7(c).

References

[52] Gillespie DT (1992) A rigorous derivation of the chemical master equation. *Physica A* 188:404–425.

[53] Wu J, Vidakovic B, Voit EO (2011) Constructing stochastic models from deterministic process equations by propensity adjustment. *BMC Systems Biology* 5:187.

[54] Barkai N, Leibler S (2000) Biological rhythms: Circadian clocks limited by noise. *Nature* 403:267–268.

[55] Nobile F, Tempone R, Webster CG (2008) A sparse grid stochastic collocation method for partial differential equations with random input data. *SIAM Journal on Numerical Analysis* 46:2309–2345.

[56] Donoho DL, et al. (2000) High-dimensional data analysis: The curses and blessings of dimensionality. *AMS Math Challenges Lecture* pp 1–32.

[57] Lynch RE, Rice JR, Thomas DH (1964) Direct solution of partial difference equations by tensor product methods. *Numerische Mathematik* 6:185–199.

[58] Kruskal JB (1977) Three-way arrays: rank and uniqueness of trilinear decompositions, with application to arithmetic complexity and statistics. *Linear Algebra and Its Applications* 18:95–138.

[59] Smith GD (1985) *Numerical solution of partial differential equations: finite difference methods* (Oxford University Press).

[60] Dolgov SV, Savostyanov DV (2013) Alternating minimal energy methods for linear systems in higher dimensions. Part I: SPD systems, available as: http://arxiv.org/abs/1301.6068

[61] Oseledets IV, Tyrtyshnikov EE (2009) Breaking the curse of dimensionality, or how to use SVD in many dimensions. *SIAM Journal on Scientific Computing* 31:3744–3759.

[62] Oseledets IV (2010) Approximation of $2^d \times 2^d$ matrices using tensor decomposition. *SIAM Journal on Matrix Analysis and Applications* 31:2130–2145.

[63] Kolda TG, Bader BW (2009) Tensor decompositions and applications. *SIAM Review* 51:455–500.

[64] Savageau MA (1971) Parameter sensitivity as a criterion for evaluating and comparing the performance of biochemical systems. *Nature* 229:542–544.
